# Retargeting phages from bacteria to human cells

**DOI:** 10.25122/jml-2024-1013

**Published:** 2024-08

**Authors:** Sergiu Chira, Ștefan Strilciuc, Dafin Fior Muresanu

**Affiliations:** 1 Research Center for Functional Genomics, Biomedicine, and Translational Medicine, Iuliu Hatieganu University of Medicine and Pharmacy, Cluj-Napoca, Romania; 2 RoNeuro Institute for Neurological Research and Diagnostic, Cluj-Napoca, Romania; 3 Department of Neurosciences, Iuliu Hatieganu University of Medicine and Pharmacy, Cluj-Napoca, Romania

## MICROBIOLOGY AND GENE THERAPY – THE CONNECTION WITHIN

Early research in microbiology set the basis for some of the most significant discoveries in the past two centuries that impacted both scientific research and medicine. The advent of antibiotics [1] and vaccines [2] transformed the course of healthcare, turning diseases once deemed deadly into treatable conditions. In the early 1970s, the discovery of restriction enzymes – bacterial proteins with catalytic functions that can recognize specific nucleotide sequences in foreign DNA – led to a new scientific era of genetic engineering. This technology allows researchers to combine DNA fragments from different sources to create new DNA molecules with new functions and characteristics. As a result, genetic engineering set the basis for gene therapy, which aims to treat health conditions by transferring a normal gene into afflicted cells using a vector to compensate for a defective counterpart [3].

Human viruses are often chosen as gene therapy vectors, as they retain millions of years of evolution, enabling them to infect cells efficiently. Despite their early success in treating severe immunodeficiency in two patients, it later became evident that these viruses can still be pathogenic. They may trigger severe immune responses or activate proto-oncogenes, leading to malignant cell transformations. In addition, their natural tropism towards a particular cell type must be altered if other cells are meant to be targeted by these recombinant viruses [4]. Consequently, the clinical use of human viruses in gene therapy remains limited, with few approved viral vectors available for directly targeting diseased cells in patients [5,6]. Most advances have been seen in immune cell therapies for malignant disorders, particularly those utilizing chimeric antigen receptor (CAR) technology [7]. Such therapies still use the ex vivo approach from the early years of gene therapy, where immune cells are harvested from the bloodstream of the patients, infected with a recombinant viral CAR construct, and then infused back into the patient’s body to achieve therapeutic effects.

## FROM PHAGE DISPLAY TO GENE THERAPY VECTORS

Phages, or bacteriophages, are viruses that specifically infect bacteria, utilizing their resources to replicate and propagate to other bacterial cells. The life cycle of phages, combined with advancements in genetic engineering, has led to the development of a technology used for decades to study protein-protein interactions and discover new targeting peptides for eukaryotic cell receptors. Through genetic engineering, random peptide sequences are displayed as fusion peptides on the coat proteins of phages, which are then multiplied in bacteria as a pool of random phage display libraries. These phages are incubated with proteins immobilized on microtiter plates or human cells cultured in these plates. Low-affinity phage display peptides are washed away, while those that show an affinity for a specific protein or cell receptor are eluted and used to infect bacteria, enriching the eluted phage-displayed peptides. This process, known as biopanning, can undergo several selection rounds until the peptide with the highest affinity for a target protein or receptor is identified. The sequence of the selected peptide is then determined by sequencing the phage DNA [8].

In 2006, a paper published in *Cell* described and characterized a novel hybrid adeno-associated virus (AAV) and phage vector (AAVP) that combines the biological proprieties of human viruses for replication in human cells with phage display technology. This vector expresses the tumor-targeting peptide RGD4C on the minor coat protein of the M13 phage, which harbors the AAV genome. Because the phages do not have receptors on the human cells, displaying the RGD4C peptide gives enhanced specificity for the tumor cells that express αV integrins. When a therapeutic gene is inserted into the phage genome, significant tumor growth reduction was observed in several mouse models of human tumors following intravenous infection [9].

Compared to conventional human virus-derived vectors, this type of phage vector offers several benefits. Using standard molecular biology facilities, they can be produced in bacteria at high titers within just three days. Lacking pathogenicity, phage-derived vectors can be administered systemically, providing a preferred route for gene therapy applications to achieve enhanced biodistribution into the target cells [10]. Additionally, these vectors can cross the blood-brain barrier, presenting new possibilities for treating brain tumors, such as the highly malignant glioblastoma [11]. Finally, phage-derived vectors do not require special cold chain facilities for storage and remain highly stable at 4°C for extended periods [10].

## TRENDS IN PHAGE-DERIVED GENE THERAPY VECTORS

This transition from peptide display to gene therapy vectors has paved the way for further research to enhance targeting capabilities and transduction efficiency. Such improvements are focused on the endosomal escape during internalization into the target cells, a limiting step for phage-derived vectors, by expressing short peptides on the coat proteins that act as proton sponges and promote endosome breakage [12]. Additionally, studies have shown that the size of phage-derived vectors impacts the transduction efficiency of target cells. Smaller vectors can diffuse more effectively into the extracellular matrix, and their intracellular trafficking is also improved [13]. Consequently, there is ongoing investigation into minimal vectors that could replace the larger phage genome, offering a more efficient delivery system.

Most phage-derived vectors display targeting peptides on the minor coat protein, which consists of only 5 copies per phage particle, compared to the major coat protein, which has approximately 2,700 copies. Expressing targeting ligands on the major coat protein has been shown to significantly enhance transduction efficiency [14]. However, the length of the displayed peptide poses a limitation, as larger peptides can impair vector titers [15]. Further investigation is needed to overcome this size constraint and enhance cellular transduction.

## Conflict of interest

The authors declare no conflict of interest.

## Acknowledgement

This work is a part of the grant number TE 66/2022, ID PN-III-P1-1.1-TE-2021-0939, Title: Development of a gene therapy vector by genetic modification of the M13 bacteriophage for high efficiency tumor targeting (ONCOPHAGE).
